# Effect of a rosmarinic acid supplemented hemodialysis fluid on inflammation of human vascular endothelial cells

**DOI:** 10.1590/1414-431X20176145

**Published:** 2017-10-19

**Authors:** W-J. Wang, M-H. Cheng, J-H. Lin, C-S. Weng

**Affiliations:** 1 Department of Biomedical Engineering, Chung Yuan Christian University, Chungli, Taiwan; 2 Division of Nephrology, Department of Internal Medicine, Taoyuan General Hospital, Ministry of Health and Welfare, Taoyuan, Taiwan; 3 Department of Rehabilitation, TaoYuan General Hospital, Ministry of Health and Welfare, Taoyuan, Taiwan

**Keywords:** Rosmarinic acid, Hemodialysis fluid, Anti-inflammation, Endothelial cells, LPS

## Abstract

Chronic systemic inflammation and repetitive damage of vascular endothelia by incompatible dialysis system are probable causes of cardiovascular disease in patients on dialysis. The present study aimed to assess *in vitro* biocompatibility and anti-inflammatory effect of hemodialysis fluid supplemented with rosmarinic acid (RA) using human umbilical vein endothelial cells (HUVEC). HUVECs (5×10^6^ cells/mL) were pre-exposed to 1 μg/mL of lipopolysaccharides (LPS) and incubated with RA-supplemented hemodialysis fluid (HDF). Cytotoxicity was assessed qualitatively by morphologic assessment and quantitatively by MTT assay. Expressions of proinflammatory mediators were assessed using quantitative real-time PCR and production of NO was quantified. Phosphorylation of AKT and nuclear localization of nuclear factor kappa B (NF-κB) were examined using western blotting. Exposure of HUVECs to RA-supplemented HDF had no influence on morphology and viability. Inhibition of proinflammatory mediator production in HUVECs by RA supplementation to HDF was significant in a dose-dependent manner. Exposure to RA-supplemented HDF resulted in a decrease in nitric oxide synthase expression and reduction of NO production in LPS-stimulated HUVECs. RA supplementation of HDF suppressed Akt activation in LPS-stimulated HUVECs. In addition, the level of cellular IκB was increased in parallel to a reduced nuclear translocation of NF-κB in LPS-induced endothelial cells. Our results suggest that RA-supplemented HDF is biocompatible and significantly suppressed inflammation induced in endothelial cells. In this respect, the use of HDF supplemented with RA could alleviate inflammation and improve long-term treatment of patients with renal failure on dialysis. Further clinical studies are required to confirm the effects.

## Introduction

Chronic kidney disease (CKD) is characterized by a slow decline in renal function and progresses to end-stage renal disease (ESRD). Kidney transplantation has been recognized as the optimal treatment for ESRD patients with prolonged survival and improved quality of life (1). However, given the increased incidence of ESRD and shortage of available organs, most patients with ESRD rely on some form of dialysis to compensate for renal insufficiency. Mortality and morbidity rates of ESRD patients on dialysis remain high due to cardiovascular diseases such as atherosclerosis (2–5). Factors contributing to vascular abnormality include hypertension, hyperglycemia, uremia, dyslipidemia and proteinuria (6,7). Recently, chronic inflammation has been considered to play a critical role in the pathogenesis of vascular disease in ESRD patients maintained on dialysis (8). It is known that levels of C-reactive protein and proinflammatory cytokines are elevated and associated with high cardiovascular mortality in ESRD patients (9). Inflammation in dialysis patients is plausibly attributed to insufficient cytokine clearance, accumulated reactive oxygen species and oxidative damage due to renal failure (10). In addition, bio-incompatibility of the dialysis system has been reported to promote chronic inflammation in patients on dialysis. Although the mechanism underlying inflammation in CKD remains unclear, modulation of pro-inflammation represents a promising strategy to manage the poor outcome of CKD.

Rosmarinic acid (α-o-caffeoyl-3, 4-dihydroxyphenyl lactic acid; RA) is a phenolic compound commonly found in Labiatae herbs such as rosemary, lemon balm, mint, perilla and sweet basil. Several studies have reported that RA possesses anti-inflammatory properties such as reducing histamine release of mast cells, blocking complement activation, and inhibiting cyclooxygenase activity (11–14). *In vivo* studies have shown that RA inhibits several complement-dependent inflammatory processes, including cobra venom factor-induced paw edema and ovalbumin/antiovalbumin-mediated passive cutaneous anaphylaxis (15,16). In addition, RA has been shown to exert an antioxidative effect by reducing liver damage induced by D-galactosamine and lipopolysaccharides in mice (17).

In the present study, we evaluated the biocompatibility of hemodialysis fluid (HDF) supplemented with RA. The effects of RA-supplemented HDF were assessed on cell viability and production of pro-inflammatory cytokines in transcriptional and translational levels. In addition, the mechanism underlying the anti-inflammatory effect of RA-supplemented HDF was elucidated.

## Material and Methods

### Chemicals and reagents

Aprotinin, leupeptin, 2′,7′-dichlorofluorescin diacetate, lipopolysaccharides (LPS), 3-(4,5-dimethylthiazol-2-yl)-2,5-diphenyl-tetrazolium bromide (MTT), Nonidet P-40, phenylmethylsulfonyl fluoride (PMSF), RA, sodium fluoride, sodium chloride, sodium phosphate, Tris-HCl, Triton X-100, and Tween-20 were purchased from Sigma-Aldrich (USA). Bicarbonate-based buffer hemodialysis concentrate No. 110 was obtained from Taiwan Biotech Co. (Taiwan). Dulbecco's-modified Eagle's medium F12 (DMEM-F12), RPMI-1640 medium and fetal bovine serum (FBS) were obtained from Gibco (USA).

### Cell culture and treatment

Human umbilical vein endothelial cells (HUVECs) were isolated from normal human umbilical cords, digested with 0.05% trypsin containing 0.02% EDTA, and eluted with DMEM-F12. Cells were grown in DMEM-F12 supplemented with 10% FBS, 100 U/mL penicillin, and 100 U/mL streptomycin. Cells were maintained in a 37°C humidified incubator with 5% CO_2_ to reach 80% confluency. Passages 2-6 were used for experiments. RA-supplemented HDF was prepared by dissolving RA in HDF. HUVECs were pretreated with 1% FBS DMEM-F12 for 16 h, and then treated with 1 μg/mL LPS in the presence or absence of RA (5–50 μM) in HDF for 30 min (immunoblot assay), 4 h (PCR and quantitative real-time PCR analysis), or 24 h (cell viability assay). Thereafter, cells were washed twice with phosphate-buffered saline (PBS), pH 7.4, for further assay.

### Cell viability

Cell viability was determined by mitochondrial-dependent reduction of MTT to formazan. Briefly, 10 μL of MTT solution (5 mg/mL in DMEM) was added to the cell supernatant and incubated for 4 h at 37°C. After removal of medium, 2-propanol was added to lyse cells and to solubilize formazan. The absorbance of formazan was measured using a microplate reader (Benchmark, Bio-Rad Laboratories, USA) at 570 nm. The absorbance of formazan generated by untreated cells was taken as 100%.

### RNA extraction, RT-PCR and quantitative real-time PCR (qPCR)

Total RNA was isolated using the RNeasy kit (Qiagen, USA) according to the manufacturer's instructions. The purified RNA was used as a template to generate first-strand cDNA synthesis using RevertAid^TM^ First Strand cDNA Synthesis Kit (Fermentas, Life Sciences, Germany). The primer sequences used for RT-PCR and qPCR are listed in Table 1. RT-PCR experiments were performed in triplicates for each sample. qPCR was performed using the ABI PRISM 7700 sequence detection system (Applied Biosystems, USA). For mRNA quantitation, FastStart Universal SYBR Green Master (Roche Applied Science, Germany) was used for Taqman PCR. The threshold cycle numbers were calculated using the ΔΔCT relative value method and normalized to GAPDH. qPCR experiments were performed in duplicates for each sample. The correct size of the PCR products was confirmed by agarose gel electrophoresis.

**Table 1.** List of primer sequences.

Target gene	
IL-1β	
Forward	5′-AACCTGCTGGTGTGTGACGTT-3′
Reverse	5′-CAGCACGAGGCTTTTTTGTT-3′
IL-6	
Forward	5′-ATGAACTCCTTCTCCACAAGCGC-3′
Reverse	5′-CAAGAGCCCTCAGGCTGGACTG-3′
iNOS	
Forward	5′-GCGGAGCGATGGGAAGCATG-3′
Reverse	5′-CCCGAGCTCCTGGAACCAC-3′
TNF-α	
Forward	5′-CACGCTCTTCTGTCTACTGA-3′
Reverse	5′-GGACTCCGTGATGTCTAAGT-3′
GAPDH	
Forward	5′-ACCACAGTCCATGCCATCAC-3′
Reverse	5′-TCCACCACCCTGTTGCTTGA-3′

### Nitric oxide assay

Nitric oxide (NO) levels in cell culture supernatant fractions were determined using the Griess reaction. In brief, Raw264.7 cells were treated with 1, 5, or 15 μg/mL PFE for 1 h, followed by incubation with 1 μg/mL LPS for 24 h. Nitrite in the culture supernatants was mixed with the same volume of Griess reagent (1% sulfanilamide and 0.1% N-[1-naphthyl]-ethylenediamine dihydrochloride in 5% phosphoric acid). Absorbance at 540 nm was measured, and the nitrite concentration was determined using sodium nitrite (NaNO_2_) as a standard.

### Subcellular fractionation

Cells were washed with PBS and incubated with a lysis buffer (10 mM HEPES, pH 7.6, containing 15 mM KCl, 2 mM MgCl_2_, 0.1 mM EDTA, 1 mM dithiothreitol, 0.05% v/v Igepal CA-630 and 1 mM PMSF, 1 mM sodium orthovanadate, 50 mM sodium fluoride, 10 μg/mL leupeptin, and 10 μg/mL aprotinin) for 10 min. Cell lysates were centrifuged at 2500 *g* for 10 min at 4°C. The supernatant containing the cytosol (cytosolic fraction) was further centrifuged at 20,000 *g* for 15 min at 4°C. The pellets containing nuclei were washed with PBS, resuspended in nuclear buffer (25 mM HEPES, pH 7.6, 0.1% v/v Igepal CA-630, 1 M KCl, 0.1 mM EDTA, 1 mM PMSF, 1 mM sodium orthovanadate, 2 mM sodium fluoride, 10 μg/mL leupeptin, and 10 μg/mL aprotinin), and centrifuged at 10,000 *g* for 15 min at 4°C. The resulting supernatant, namely nuclear fraction, was collected.

### Immunoblot assay

Cells (5×10^5^ cells/mL) were harvested, washed twice with ice-cold PBS, and lysed in a lysis buffer (50 mM Tris-HCl, pH 7.5, 150 mM NaCl, 1% v/v Igepal CA-630, 1 mM PMSF, 1 mM sodium fluoride, and 10 μg/mL aprotinin and leupeptin). The cell lysates were centrifuged at 14,000 *g* for 10 min at 4°C to remove debris. The supernatants were collected and crude protein concentrations were determined using BCA^TM^ protein assay kit (Pierce, USA). Crude proteins (30 μg per lane) were electrophoresed on 12.5% SDS-polyacrylamide gel, and transferred onto a nitrocellulose membrane (Millipore, USA). The blotted membrane was blocked with 5% w/v skim milk in PBS, and then incubated for 2 h with 1/1000 dilution of the specific antibodies against phosphorylated AKT (pAKT), AKT, phosphorylated IκB-α (pIκB-α), NF-κB, Histone H1 (Cell Signaling Technologies, USA) or glyceraldehyde 3-phosphate dehydrogenase (GAPDH; Abcam, UK). Bound antibodies were detected using 1/2000 dilution of peroxidase-conjugated secondary antibodies (Abcam) and ECL chemiluminescence reagent (Millipore) as the substrate system.

### Statistical analysis

Data are reported as means±SD of three independent experiments. Statistical comparisons were made by one-way analysis of variance (ANOVA), followed by Duncan’s multiple-comparison test. Student's *t*-test was employed to determine the significance of the difference between two groups. Differences were considered significant when P values were <0.05.

## Results

### Cytotoxicity of RA-supplemented HDF

**Figure 1.**
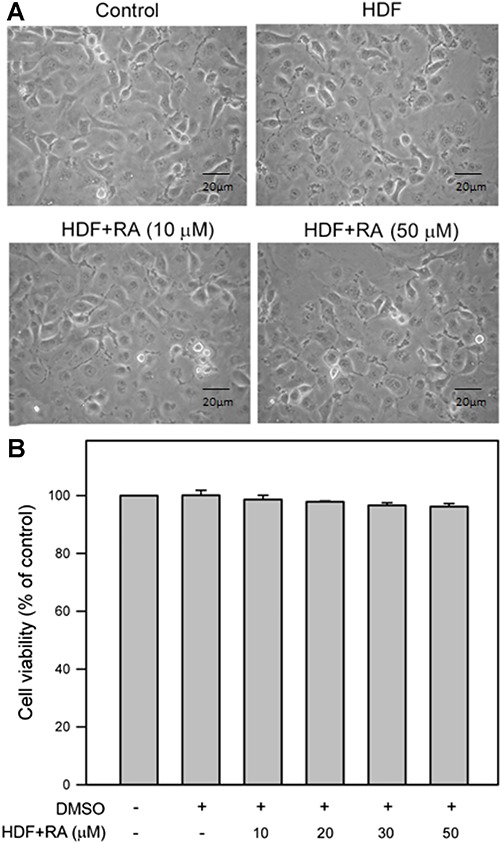
Effects of hemodialysis fluid (HDF) supplemented with rosmarinic acid (RA) on morphology and viability of human umbilical vein endothelial cells. *A*, Cells were treated with HDF or HDF supplemented with 10 mM or 50 mM RA for 24 h, and the cell morphology was monitored by phase-contrast microscopy (400×). *B*, Cells were treated with DMSO, HDF or HDF supplemented with serial concentrations of RA for 24 h, and the cell viability was determined by MTT assay. These results revealed that HDF supplemented with RA (≤50 μM) had no significant influence on morphology and cell viability of HUVECs.

As shown in Figure 1A, HUVECs were incubated with HDF supplemented with a serial concentration of RA (0, 10, and 50 μM) for 24 h, and the morphology of treated HUVECs was slightly affected. Additionally, cytotoxicity was not significant compared to controls (Figure 1B). These results revealed that HDF supplemented with RA (≤50 μM) had no significant influence on morphology and cell viability of HUVECs.

### RA-supplemented HDF inhibited proinflammatory mediator production

**Figure 2.**
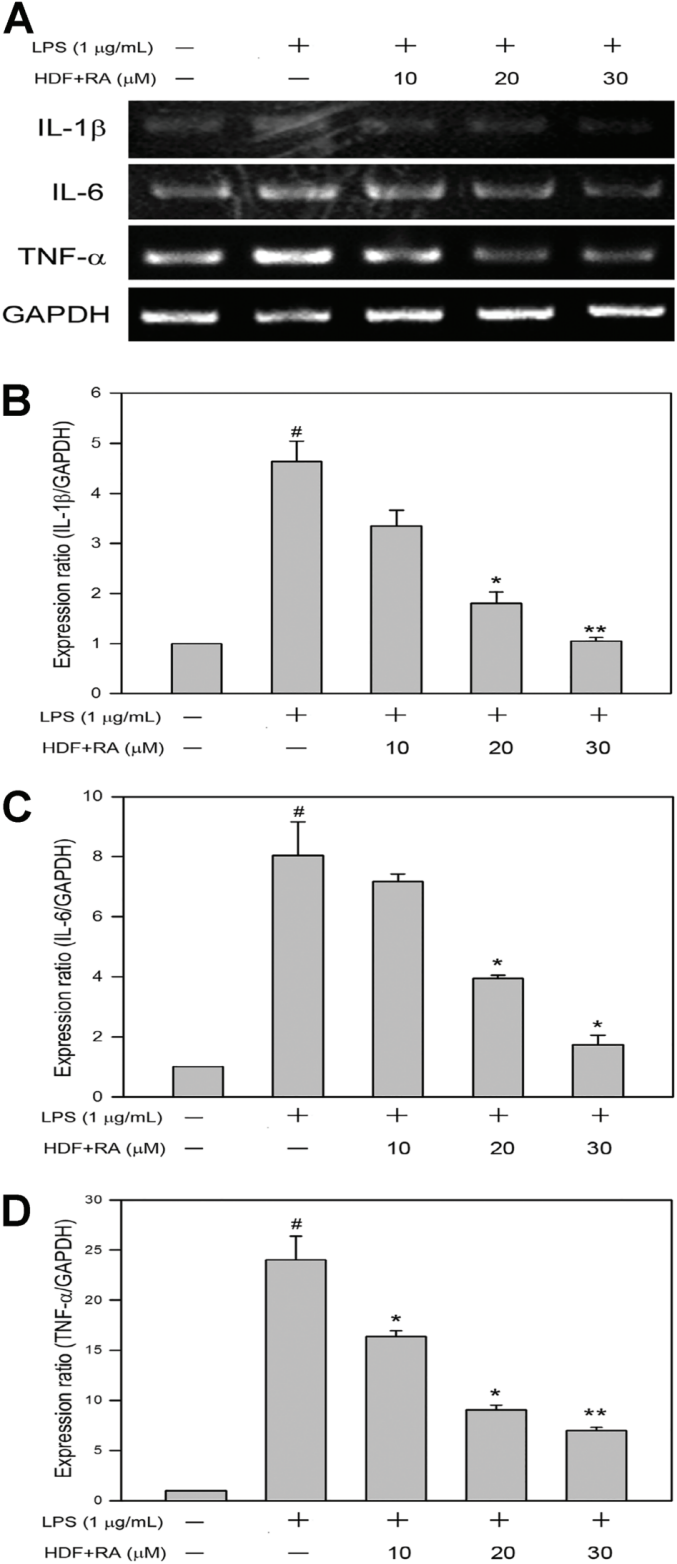
Hemodialysis fluid (HDF) supplemented with rosmarinic acid (RA) inhibited mRNA expression of pro-inflammatory cytokines in LPS-stimulated human umbilical vein endothelial cells. Detection and quantitation of IL-1β, IL-6 and TNF-α were done by *A*, RT-PCR, and *B*-*D*, q-PCR. Data are reported as means±SD. Three independent experiments were performed for quantitative and statistical analysis. ^#^P<0.05 compared to control; *P<0.05 and **P<0.005 compared to LPS alone (ANOVA).

Endotoxin is a robust stimulator to activate vascular endothelium, consequently leading to systemic inflammatory responses (18). Therefore, effects of RA-supplemented HDF on LPS-induced mRNA expression of inflammatory mediators in HUVECs were investigated. As shown in Figure 2A, mRNA expressions of IL-1β, IL-6 and TNF-α in HUVECs were significantly increased in response to the presence of LPS, and reduced in the presence of RA-supplemented HDF in a dose-dependent manner. Quantitative analysis using q-PCR revealed that HDF supplemented with 30 μM RA suppressed average mRNA expression of IL-1β, IL-6 and TNF-α to 25.6, 24.4, and 30.4% of LPS-alone, respectively (P<0.05; Figure 2B-D). These results showed that RA-supplemented HDF was capable of significantly reducing LPS-induced mRNA expression of IL-1β, IL-6, and TNF-α in HUVECs.

### RA-supplemented HDF inhibited mRNA expression of iNOS and the consequent NO production

**Figure 3.**
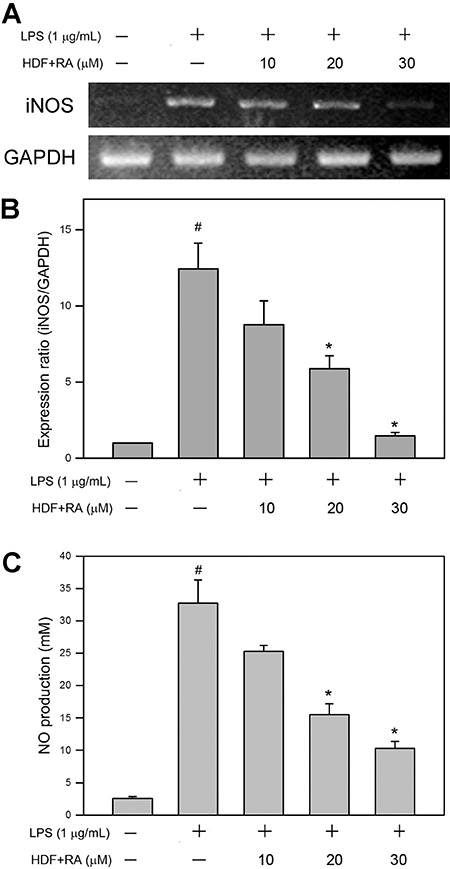
Hemodialysis fluid (HDF) supplemented with rosmarinic acid (RA) suppressed mRNA expression of inducible nitric oxide synthase (iNOS) and nitric oxide (NO) production by lipopolysaccharides (LPS)-stimulated human umbilical vein endothelial cells. Detection and quantitation of iNOS were done by RT-PCR (*A*), and q-PCR (*B*). The culture medium after the treatment was collected for NO assay. The concentration of NO was determined by Griess reagent using NaNO_2_ as standard (*C*). Data are reported as means±SD. Three independent experiments were performed for quantitative and statistical analysis. ^#^P<0.05 compared to control; *P<0.05 compared to LPS alone (ANOVA).

NO, synthesized by inducible nitric oxide synthase (iNOS), is an important inflammatory mediator secreted by LPS-stimulated endothelial cells (19). Therefore, effects of RA-supplemented HDF on mRNA expression of iNOS and NO production by HUVECs were investigated. As shown in Figure 3A, mRNA expression level of iNOS in HUVECs was significantly elevated by LPS, and diminished by HDF supplemented with serial concentrations of RA (10, 20, and 30 μM) in a dose-dependent manner. Moreover, quantitative analysis using q-PCR revealed that HDF supplemented with 30 μM RA suppressed mRNA expression of iNOS to 20.8% of TNF-α alone (P<0.05; Figure 3B). NO production of LPS-stimulated HUVECs was also determined. As shown in Figure 3C, exposure of HUVECs to LPS resulted in a significant increase in NO production, and the level of NO induced by LPS was suppressed by HDF supplemented with serial concentrations of RA (10, 20, and 30 μM) in a dose-dependent manner. Quantitative analysis using sodium nitrite as standard revealed that HDF supplemented with 30 μM RA suppressed NO production to 20.8% of LPS alone (P<0.05; Figure 3C). These findings revealed that RA-supplemented HDF significantly inhibited LPS-induced iNOS expression and the consequent NO production by HUVECs.

### RA-supplemented HDF suppressed phosphorylation of AKT and IκB-α and nuclear translocation of NF-κB in LPS-stimulated HUVECs

**Figure 4.**
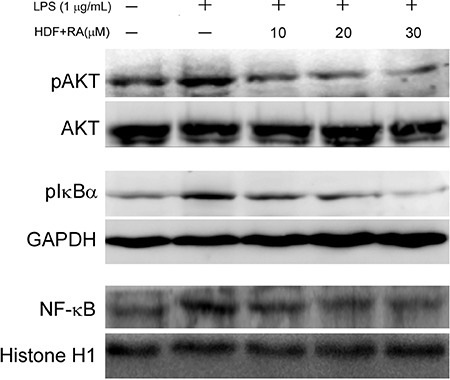
Hemodialysis fluid (HDF) supplemented with rosmarinic acid (RA) increased phosphorylation of cytosolic AKT and IκBα and diminished nuclear NF-κB in lipopolysaccharides (LPS)-stimulated human umbilical vein endothelial cells. Phosphorylation of cytosolic AKT and IκB-α, and the level of nuclear NF-κB were determined by immunoblot using specific antibodies and chemiluminescence development. Levels of GAPDH and histone H1 were used as cytosolic and nuclear control.

Activation of AKT and the subsequent nuclear translocation of transcription factor NF-κB play important roles in production of proinflammatory cytokines. Therefore, effects of RA-supplemented HDF on phosphorylation of AKT and nuclear translocation of NF-κB in LPS-stimulated HUVECs were determined. As shown in Figure 4, exposure to LPS led to significantly increased phosphorylation of AKT in HUVECs, and the phosphorylation of AKT induced by LPS was decreased with the presence of HDF supplemented with RA (10, 20, and 30 μM) in a dose-dependent manner. In addition, phosphorylation of IκB-α and NF-κB inhibitor, concomitantly with nuclear translocation of NF-κB, was increased in LPS-stimulated HUVECs. The increases of both IκB-α phosphorylation and NF-κB nuclear translocation were inhibited by HDF supplemented with RA (10, 20, and 30 μM) in a dose-dependent manner. These findings revealed that HDF supplemented with RA was capable of attenuating LPS-induced AKT activation, IκB-α phosphorylation and NF-κB translocation.

## Discussion

For patients with CKD on hemodialysis, endotoxins come not only from the gut, but also from impure dialysate, which contains endotoxin derived from environmental bacteria. Even a low level of endotoxemia constitutes chronic inflammation, which has been proposed to play a major role in the development of cardiovascular diseases and mortality among patients with renal failure (20,21). A recent study also suggests that vein endothelial cells may play important roles in the pathophysiology of systemic inflammation-associated diseases such as sepsis and septic cardiomyopathy (22). Therefore, diminishing exposure to endotoxin and suppressing endotoxin-induced chronic inflammation are important and urgent for the patients on hemodialysis.

Circulating LPS in blood contributes to vascular inflammation through the activation of endothelial cells and vascular smooth muscular cells (23). Endotoxin concentration in hemolysis patients with systemic inflammation is suggested to be as low as 1 EU/mL. Previous studies have reported that IL-1β, IL-6 and TNF-α are over-expressed in HUVEC exposed to LPS (22,24,25). IL-6 possesses various biological properties in a number of chronic endothelial dysfunctions such as modulation of hematopoiesis, proliferation and differentiation of lymphocytes, and induction of acute-phase reactions (26). In addition, both IL-1β and IL-6 are also known as key mediators in chronic inflammation and are associated with long-term hemodialysis complications such as erythropoietin hyporesponsiveness, cardiovascular morbidities and amyloidosis-related bone disease (27,28). In addition to environmental factors such as LPS, genomic research has highlighted the contribution of non-environmental factors to the development of arthrosclerosis in hemodialysis patients (29,30). In the present study, we employed HUVEC as a cell model to explore the effectiveness of RA supplementation in HDF on LPS (1 μg/mL) induced inflammatory responses. Our findings confirmed that IL-1β, IL-6, and TNF-α were over-expressed in HUVEC exposure to LPS and demonstrated that HDF supplemented with RA significantly inhibited over-expression of IL-1β, IL-6, and TNF-α in LPS-stimulated HUVECs. According to the experimental setting, it is suggested that RA supplement has a potential to alleviate chronic inflammation of endothelial cells induced by endotoxin even up to a concentration of 1 μg/mL. However, further studies *in vivo* and in the clinical setting are required to explore the effectiveness of RA-supplemented HDF on reducing hemodialysis-associated inflammation. Moreover, *in vivo* studies for toxicological risk assessment of RA are critically necessary.

It is well known that endotoxemia leads to excessive production of the vasodilator NO via iNOS (31,32). The highly elevated NO production further causes downregulation of endothelial NOS (eNOS), leading to an attenuated endothelium-dependent vasodilatory response (33). Consistently, over-expression of iNOS and elevation of NO production were detected in HUVECs treated with LPS. The over-expression of iNOS and elevation of NO production in LPS-treated HUVECs were significantly inhibited by RA-supplemented HDF. However, given the complexity of vasculature, further animal studies are required to confirm the findings obtained using HUVEC cell model and to elucidate the responses of lymphocytes and leukocytes to RA-supplemented HDF in vascular tissues.

Activation of AKT and the subsequent nuclear translocation of transcription factor NF-κB play important roles in production of proinflammatory cytokines (34). NF-κB is the primary transcription factor mainly involved in regulating inflammatory and immune responses to extracellular stimulus. Upon activation, inhibitory protein IκB-α is rapidly phosphorylated and attributed to nuclear translocation of NF-κB (p65), which binds to related DNA elements and activates its target genes such as IL-1β and IL-6 (35). To investigate the inhibitory mechanism of IL-1β and IL-6 expression in HUVECs, we examined the effect of RA-supplemented HDF on IκB-α phosphorylation and nuclear NF-κB. Our findings showed that the levels of either IκB-α phosphorylation or nuclear NF-κB (p65) were markedly decreased in LPS-induced HUVECs as a result of RA supplementation, suggesting that this is mediated through its influence on IκB-α phosphorylation, thereby decreasing the nuclear translocation of NF-κB.

In conclusion, we demonstrated that HDF supplemented with RA did not affect morphology or viability of human endothelial cells and exerted inhibitory effects on LPS-induced inflammatory responses, including expression of IL-1β, IL-6, TNF-α, and iNOS, as well as NO production. These results suggest that RA-supplemented HDF is biocompatible and that RA significantly inhibits upregulation of inflammatory mediators induced by LPS, suggesting that RA is a potential supplement for most patients on dialysis. Further *in vivo* studies are necessary to examine the usefulness of RA-supplemented HDF in clinical practice.
